# Distribution of melanopsin positive neurons in pigmented and albino mice: evidence for melanopsin interneurons in the mouse retina

**DOI:** 10.3389/fnana.2014.00131

**Published:** 2014-11-20

**Authors:** Francisco J. Valiente-Soriano, Diego García-Ayuso, Arturo Ortín-Martínez, Manuel Jiménez-López, Caridad Galindo-Romero, Maria Paz Villegas-Pérez, Marta Agudo-Barriuso, Anthony A. Vugler, Manuel Vidal-Sanz

**Affiliations:** ^1^Departamento de Oftalmología, Facultad de Medicina, Campus de Espinardo, Universidad de Murcia, e Instituto Murciano de Investigación Biosanitaria-Hospital Clínico Universitario Virgen de la Arrixaca (IMIB-ARRIXACA)Murcia, Spain; ^2^Department of Ocular Biology and Therapeutics, UCL-Institute of OphthalmologyLondon, UK

**Keywords:** melanopsin retinal ganglion cells, retinal topography, ciliary marginal zone, interneuron, displaced intrinsically photosensitive retinal ganglion cells, Brn3a, OHSt-tracing, spatial distribution

## Abstract

Here we have studied the population of intrinsically photosensitive retinal ganglion cells (ipRGCs) in adult pigmented and albino mice. Our data show that although pigmented (C57Bl/6) and albino (Swiss) mice have a similar total number of ipRGCs, their distribution is slightly different: while in pigmented mice ipRGCs are more abundant in the temporal retina, in albinos the ipRGCs are more abundant in superior retina. In both strains, ipRGCs are located in the retinal periphery, in the areas of lower Brn3a^+^RGC density. Both strains also contain displaced ipRGCs (d-ipRGCs) in the inner nuclear layer (INL) that account for 14% of total ipRGCs in pigmented mice and 5% in albinos. Tracing from both superior colliculli shows that 98% (pigmented) and 97% (albino) of the total ipRGCs, become retrogradely labeled, while double immunodetection of melanopsin and Brn3a confirms that few ipRGCs express this transcription factor in mice. Rather surprisingly, application of a retrograde tracer to the optic nerve (ON) labels all ipRGCs, except for a sub-population of the d-ipRGCs (14% in pigmented and 28% in albino, respectively) and melanopsin positive cells residing in the ciliary marginal zone (CMZ) of the retina. In the CMZ, between 20% (pigmented) and 24% (albino) of the melanopsin positive cells are unlabeled by the tracer and we suggest that this may be because they fail to send an axon into the ON. As such, this study provides the first evidence for a population of melanopsin interneurons in the mammalian retina.

## Introduction

In the mammalian retina, melanopsin is expressed by a subset of intrinsically photosensitive retinal ganglion cells (ipRGCs), which also receive synaptic input from rods and cones (Dacey et al., 2005; Schmidt et al., 2008; Weng et al., 2013). These ipRGCs are required for non-image forming vision in mice (Guler et al., 2008) and are thought to integrate rod and cone signals with their own melanopsin-driven light responses to control important aspects of circadian behavior (Panda et al., 2002), pupil constriction (Lucas et al., 2003) and most recently, image forming vision (Brown et al., 2010, 2012; Ecker et al., 2010; Estevez et al., 2012; Allen et al., 2014; Schmidt et al., 2014a).

In mice, ipRGCs are a heterogeneous population, with at least five different subtypes described to date (Berson et al., 2010; Ecker et al., 2010; Jain et al., 2012; Karnas et al., 2013). The subtypes M1-M4 can be identified using immunohistochemistry for melanopsin (Berson et al., 2010; Estevez et al., 2012), while M5 cells have only been identified using *Opn4.Cre* reporter mice (Ecker et al., 2010; Hughes et al., 2013). In terms of the topographic distribution of ipRGCs, it is known that in both rats (Hannibal et al., 2002; Hattar et al., 2002; Vugler et al., 2008; Galindo-Romero et al., 2013; Nadal-Nicolás et al., 2014) and mice (Hughes et al., 2013), the majority of melanopsin positive cells are located in dorsal retina with a melanopsin-rich plexus in the ciliary marginal zone (CMZ) of both species (Vugler et al., 2008; Semo et al., 2014). Topography of ipRGCs has also been shown to be an important factor in determining the spectral properties of their cone-based input (Estevez et al., 2012; Hughes et al., 2013).

Previous work from our laboratory has shown that rodents possess a horizontal visual streak of high RGC density just dorsal to the optic nerve (ON; Salinas-Navarro et al., 2009b,c; Ortín-Martínez et al., 2010, 2014) and that, in rats, ipRGCs can be found at highest density just peripheral to this structure (Galindo-Romero et al., 2013; Nadal-Nicolás et al., 2014). Given the importance of mouse models in melanopsin research, we therefore decided to undertake a detailed study of ipRGC distribution in pigmented and albino mice, with reference to the general RGC population (as labeled by retrograde tracer and or Brn3a). The comparison between pigmented and albino mice is of particular importance given the use of albino rodents to study the melanopsin system (Hattar et al., 2002; Gooley et al., 2003; Hannibal et al., 2005; González-Menéndez et al., 2010; Xue et al., 2011; Esquiva et al., 2013).

Because melanopsin expression stops at the ON head, to date, most of our knowledge concerning the retinofugal projection of ipRGCs in mice comes from *tau-lacZ* and *Opn4.Cre* reporter mice (Hattar et al., 2006; Brown et al., 2010; Ecker et al., 2010; Chen et al., 2011), with only a few studies employing classical retrograde tracing techniques (Viney et al., 2007; Baver et al., 2008). The general opinion from these studies is that Brn3b negative M1 cells project to the suprachiasmatic nucleus (SCN), while other ipRGC subtypes project variably to the olivary pretectal nucleus (OPN), dorsal lateral geniculate nucleus (dLGN) and superior colliculus (SC; Chen et al., 2011; Schmidt et al., 2011).

In rodents, the vast majority of RGCs project to the SC (Linden and Perry, 1983; Hofbauer and Dräger, 1985; Salinas-Navarro et al., 2009b,c; Nadal-Nicolás et al., 2014), an important structure for controlling gaze-movements of the eyes/head and integrating multimodal sensory information to initiate target location and avoidance movements (May, 2006). In order to examine what proportion of ipRGCs project to the SC in mice, we have labeled this structure by applying hydroxystilbamidine methanesulfonate (OHSt), a tracer that is actively transported in the retrograde direction from both superior colliculi (SCi).

Given recent findings that Brn3b negative M1 ipRGCs send retino-ciliary projections beyond the retinal boundary (Semo et al., 2014), we were also interested to determine if these cells send axons towards the brain, and to this end we retrogradely labeled the entire retinofugal projection from the ON. In addition to the CMZ population, we also studied the distribution of displaced ipRGCs (d-ipRGCs), the soma of which resides in the inner nuclear layer (INL). These cells are abundant in primate (Dacey et al., 2005; Jusuf et al., 2007) and rat (Nadal-Nicolás et al., 2014) retina but in mouse, their topographic distribution remains unknown (Berson et al., 2010; Jain et al., 2012; Karnas et al., 2013).

## Materials and methods

### Animal handling

Two-month-old female adult pigmented C57BL/6 (*n* = 17; 25–30 g) and albino Swiss (*n* = 15; 30–35 g) mice were obtained from the University of Murcia breeding colony. They were housed in a 12 h light 12 h dark light cycle with lights on at 08:00 and off at 20:00. Animal manipulations were carried out following the Spanish and European Union regulations for the use of animals in research (Council Directive 86/609/EEC), the ARVO statement for the use of animals in ophthalmic and vision research, and were approved by the Ethical and Animal Studies Committee of the University of Murcia (Murcia, Spain). For surgical manipulations, mice were anesthetized with an intraperitoneal (i.p.) injection of ketamine (70 mg/kg Ketolar^®^, Pfizer, Alcobendas, Madrid, Spain) and xylazine (10 mg/kg Rompun^®^, Bayer, Kiel, Germany). To prevent corneal desiccation eyes were covered with an ocular ointment (Tobrex; Alcon S. A., Barcelona, Spain). All animals were sacrificed with an overdose of sodium pentobarbital (Dolethal^®^ Vetoquinol, S.A., Especialidades Veterinarias, S.A., Alcobendas, Madrid, Spain). The experimental design is summarized in Table 1.

**Table 1:** **Experimental design**.

ipRGC population (2 month old mice)	C57BL/6	Swiss
	Pigmented	Albino
Untouched retinas: Number and distribution of ipRGCs vs. the general RGC population. Number of ipRGCs that express Brn3a.	16	11
Tracing from the optic nerve: Do all melanopsin^+^cells project their axons through the optic nerve?	12	8
Tracing from both superior colliculi: What proportion of ipRGCs become retrogradely labeled from the SCi?	7	10

*Number of retinas analyzed per group and strain. SCi: superior colliculi*.

### Tracing from the superior colliculi or from the optic nerve stump

To trace from both SCi, OHSt (Molecular Probes, Leiden, Netherlands) diluted at 10% in 0.9% NaCl and 10% dimethylsulfoxide was applied to both SCi 1 week before processing, as previously described (Villegas-Pérez et al., 1996; Salinas-Navarro et al., 2009b). To trace from the ON, 3 days before processing, a small piece of gelatine sponge (Espongostan Film, Ferrosan A/S, Denmark) soaked in OHSt diluted at 6% in the same solution as above was applied to the ocular stump of the intraorbitally transected ON of both eyes, as previously described (Vidal-Sanz et al., 1988; Lafuente López-Herrera et al., 2002; Salinas-Navarro et al., 2009b).

### Tissue processing

All animals were euthanized between 10:00 and 12:00 in order to avoid the diurnal fluctuations in melanopsin expression reported by others (González-Menéndez et al., 2009; Hannibal et al., 2013). Mice were then perfused transcardially with 4% paraformaldehyde in 0.1 M phosphate buffer. Special care was taken to maintain the orientation of the eyes: (i) after anesthesia and before perfusion a suture was placed on the superior pole of each eye; (ii) the rectus muscle insertion into the superior part of the eye and the nasal caruncle were used as additional landmarks (Salinas-Navarro et al., 2009b,c). Both retinas were dissected and prepared as flattened whole-mounts by making four radial cuts (the deepest one in the superior pole) as previously described in detail (Salinas-Navarro et al., 2009b; Galindo-Romero et al., 2011, 2013).

For cross-sectional analysis, eyes from pigmented mice that had been labeled with OHSt applied to both SCi for 1 week (*n* = 4) or to the ON for 3 days (*n* = 2), respectively, were enucleated and immunostained for melanopsin following previously described methods (Vugler et al., 2008; Ortín-Martínez et al., 2010; Galindo-Romero et al., 2011).

### Immunohistofluorescence

In all retinas, melanopsin and Brn3a were double immunodetected following previously described methods with established immunodetection protocols for retinal flatmounts where the vitreous is thoroughly cleaned before antibody incubation (Vugler et al., 2008; Nadal-Nicolás et al., 2009, 2012, 2014; Galindo-Romero et al., 2011, 2013). The primary antibody used to detect ipRGCs was the rabbit anti-melanopsin antibody UF006 (1:5000, AB-N38, Advance Targeting Systems, Thermo Scientific, Madrid, Spain). The general RGC population was detected using goat anti-Brn3a (1:500, C-20, Santa Cruz Biotechnologies Heidelberg, Germany) (Nadal-Nicolás et al., 2009, 2012; Galindo-Romero et al., 2011, 2013). The primary antibodies were then detected with the appropriate combination of fluorescently conjugated secondary antibodies: donkey anti-rabbit Alexa 594 and donkey anti-goat Alexa-488 (all diluted 1:500 and from Molecular Probes, Invitrogen, Barcelona, Spain).

From previous studies in mice, rabbit polyclonal anti-melanopsin has been shown to detect 90% of ipRGCs in *Opn4^Cre^;Z/EG* reporter mice (Brown et al., 2010) and the UF006 antibody will recognize all M1 and M2 type ipRGCs in mice, together with some larger (possibly M4 type) ipRGCs (Karnas et al., 2013).

### Image analysis

Whole mounted retinas were analyzed for melanopsin, Brn3a and OHSt signals. To reconstruct retinal whole-mounts, retinal photographs were taken following previously described procedures that are standard in our laboratory (Salinas-Navarro et al., 2009a; Cuenca et al., 2010; Galindo-Romero et al., 2011, 2013; Sánchez-Migallón et al., 2011; Vidal-Sanz et al., 2012).

In brief, using an epifluorescence microscope (Axioscop 2 Plus; Zeiss Mikroskopie, Jena, Germany) equipped with a computer-driven motorized stage (ProScan H128 Series; Prior Scientific Instruments, Cambridge, UK) controlled by image analysis software (Image-Pro Plus, IPP 5.1 for Windows; Media Cybernetics, Silver Spring, MD, USA), retinal multiframe acquisitions were photographed in a raster-scan pattern in which frames were captured side-by-side with no gap or overlap between them with a 20× objective (Plan-Neofluar, 20×/0.50; Zeiss Mikroskopie, Jena, Germany). Single frames were focused manually (see below) before the capture of each image, which was then fed into the IPP image analysis program. The scan area covers the entire retina, with a frame size of 0.2161 mm^2^/image in the mouse retina each at a resolution of 300 dots per inch. Reconstructed wholemounts were made up of 154 individual frames.

### Automated quantification and spatial distribution of Brn3a^+^RGCs

Brn3a^+^RGCs and RGCs traced from the SCi were automatically quantified following previously described methods that have been developed by our group (Salinas-Navarro et al., 2009b; Galindo-Romero et al., 2011, 2013; García-Ayuso et al., 2014). Briefly, the individual fluorescent images taken for each retinal whole-mount were processed by a specific subroutine using the IPP macro language. After quantification, Brn3a^+^RGCs isodensity maps were constructed through a quadrant analysis as previously described in detail (Galindo-Romero et al., 2011, 2013).

### Quantification, spatial distribution and soma diameter of ipRGCs

Intrinsically photosensitive retinal ganglion cells in the RGC layer were manually dotted on the retinal photomontage using the graphic editing program Adobe Photoshop CS 8.0.1 (Adobe Systems, Inc., San Jose, CA, USA). Dots were automatically quantified and their retinal location was obtained using a modified IPP macro language routine as described (Galindo-Romero et al., 2013; Nadal-Nicolás et al., 2014). In brief: after marking the ON as a reference point and drawing the retinal contour, the retinal area and the number of dots representing ipRGCs and their *x, y* spatial location were exported to a spreadsheet (Office Excel 2000; Microsoft Corp., Redmond, WA, USA).

Intrinsically photosensitive retinal ganglion cell spatial distribution was studied using the k-nearest neighbor algorithm using a Java (Oracle Corporation, Redwood Shores, California, USA) application, as described (Galindo-Romero et al., 2013; Nadal-Nicolás et al., 2014). Briefly, the user fixed the radius of the study (0.165 mm) and imported the previously obtained spread sheet with the spatial information of the ipRGCs. Those cells within the fixed radius were counted as neighbors. Spatial information was used to plot every ipRGC, and the number of neighbors served to color each ipRGC within a color scale from purple (0–1 neighbors) to red (11 or more neighbors). Data gathered after the spatial analysis allowed as well, the extraction of the number of ipRGCs at a given distance from the ON in the whole retina and in each retinal quadrant. These data were subsequently represented in bar graphs (number of cells against distance from the ON). All plots were performed using SigmaPlot (SigmaPlot^®^ 9.0 for Windows^®^; Systat Software, Inc., Richmond, CA, USA).

The method to count d-ipRGCs was similar to above. In brief, d-ipRGCs were studied in retinas traced from the ON and in these retinas Brn3a and melanopsin were double immunodetected. To investigate d-ipRGCs, each retina was photographed focusing on the INL. Each frame was focused first on the GCL, then the focus was changed to the INL and finally the image for the melanopsin signal in the INL was acquired. As reference, we used RGCs (traced and also Brn3a immunodetected) that were present in the GCL and also in the INL. After photographing the whole retina, all the frames focused on the INL were tiled as a photomontage.

For the CMZ counts, ipRGCs were included in the CMZ if they fell within ~100 µm of the retinal edge. Again, this population could be distinguished into orthotopic ipRGCs residing in the RGC layer and d-ipRGCs, which were displaced into the INL.

In addition, maps representing the distribution of ipRGCs from three superimposed retinas (normalized maps) as previously described by Nadal-Nicolás et al. (2014) (Figure 5). This was feasible because the retinas were of similar size, equally oriented and the maps were centered on the ON.

**Figure 1:**
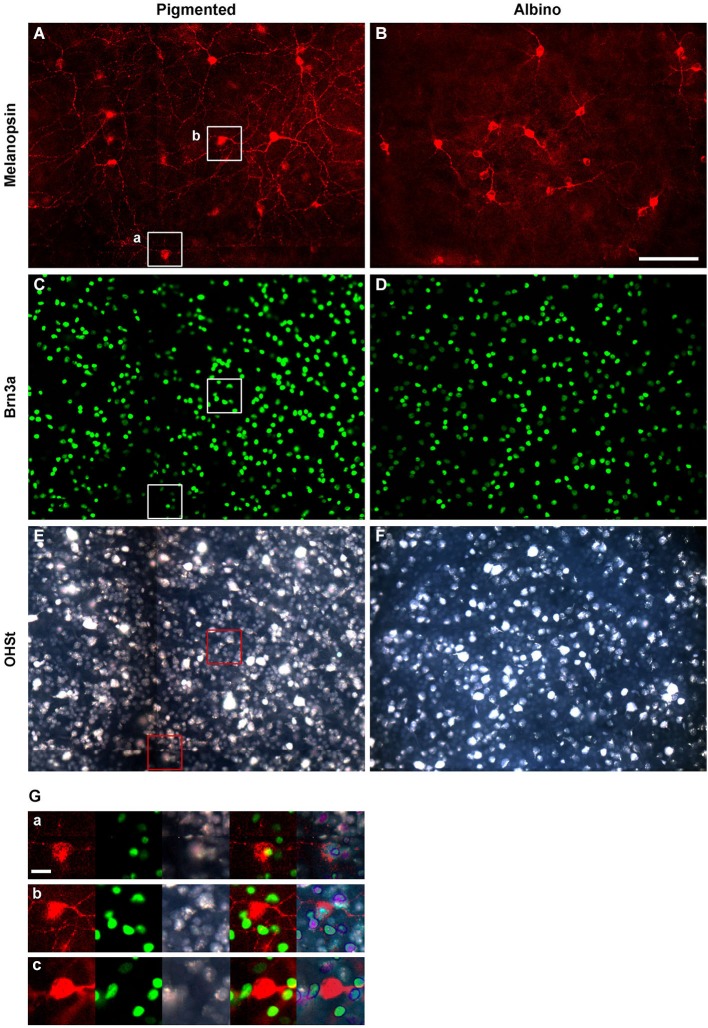
**Identification of ipRGCs in retinal whole-mounts traced from the superior colliculus**. Melanopsin **(A,B)**, Brn3a **(C,D)** and OHSt **(E,F)** signals acquired from the same frames of pigmented (left column) and albino (right column) whole-mounted retinae. **(G)** Magnifications showing co-localization between melanopsin, Brn3a and OHSt **(a)**; co-localization between melanopsin and OHSt **(b)**; and a melanopsin cell which does not co-localize with Brn3a nor OHSt **(c)**. **(a, b)** images are taken from their respective squares in **(A), (C)** and **(E)**. Scale bar in **(B)** 100 µm in **(G)** 20 µm.

**Figure 2:**
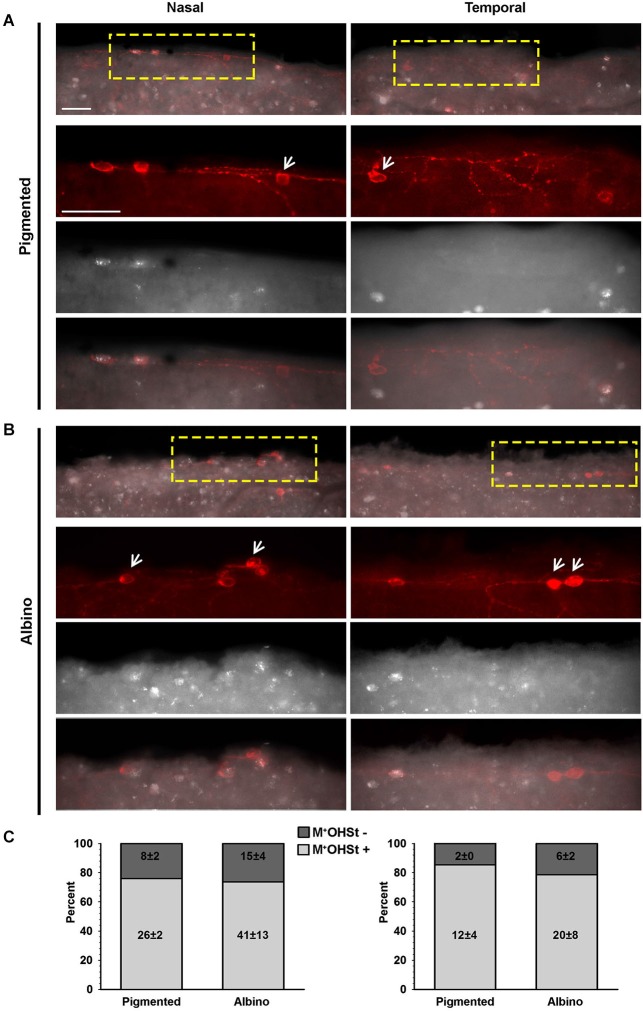
**OHSt tracing from the optic nerve reveals that not all melanopsin cells in the CMZ are ipRGCs. (A,B)** Images acquired from the nasal (left column) and temporal (right column) retinal CMZ from pigmented **(A)** and albino **(B)** animals showing melanopsin signal (red) and OHSt tracing (white). Images in the top row of **(A)** and **(B)** show a yellow region of interest which is magnified below. The arrows in **(A)** and **(B)** point to melanopsin positive (M^+^) cells which fail to retrogradely label with OHSt (M^+^OHSt^−^). **(C)** Stack bar graphs showing the percentage of M^+^ cells that were traced (OHSt^+^) or not (OHSt^−^), with 100% representing the total number of melanopsin^+^cells counted in each hemiretinal CMZ (*n* = 3 retinae per strain). Inside each bar is shown the total number (mean ± SD) of M^+^ cells found in the retinal rim of each strain in the nasal (left) or temporal (right) retina. Scale bar: 100 µm.

**Figure 3:**
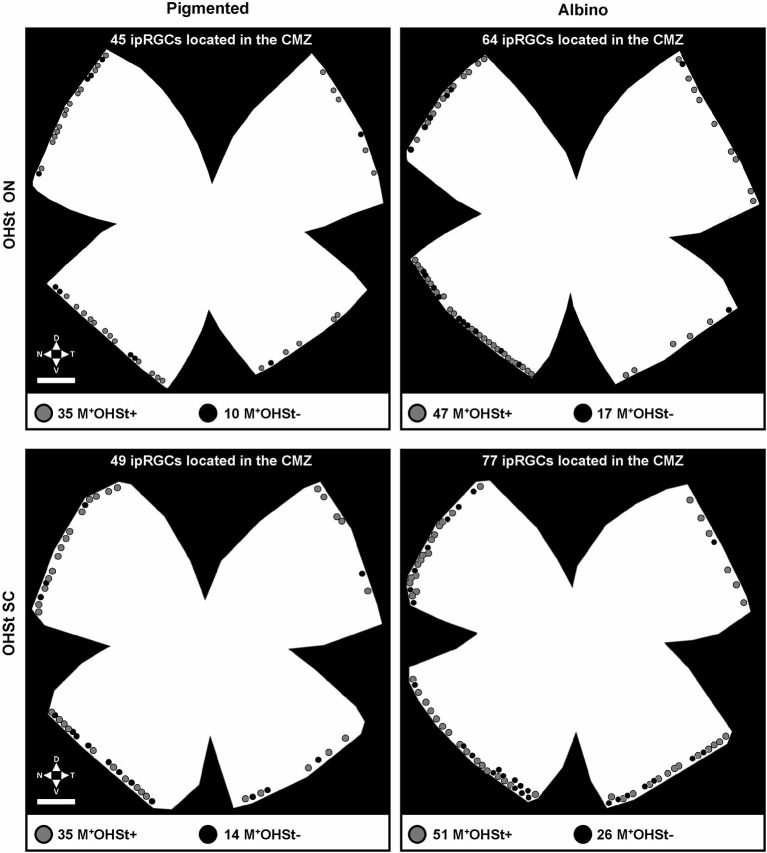
**Representative plots of melanopsin positive cells in the CMZ of pigmented and albino mice traced from either the ON or SCi**. Note the predisposition for M^+^ cells in the nasal hemiretinal CMZ of both strains. The gray dots indicate classical ipRGCs (M^+^OHSt^+^), while the black dots represent melanopsin cells which fail to retrogradely label with OHSt (M^+^OHSt^−^). Abbreviations: optic nerve (ON), superior colliculi (SCi). Scale bar: 500 µm.

**Figure 4:**
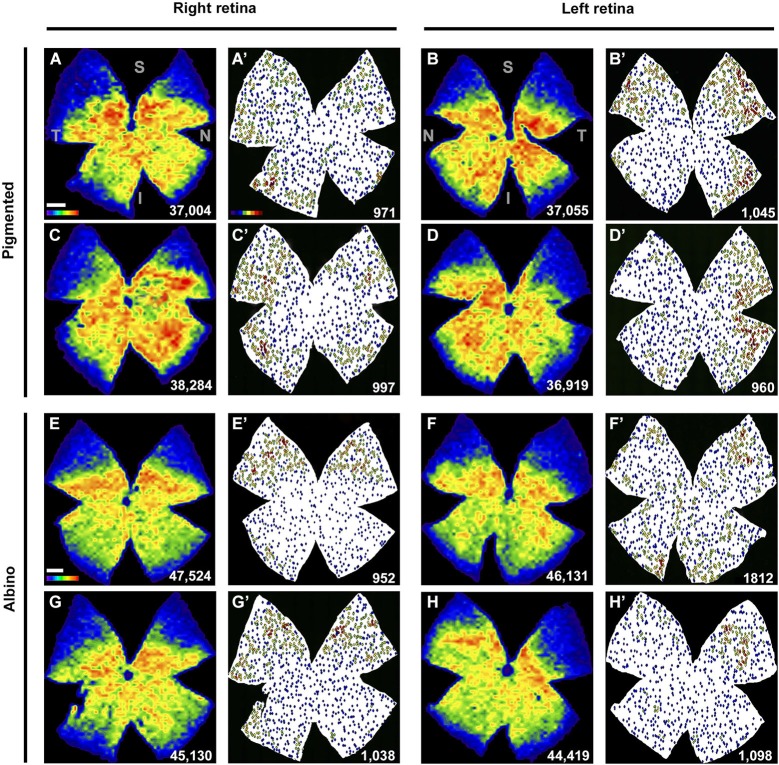
**Retinal distribution of Brn3a^+^RGCs and ipRGCs. (A–H)** Isodensity maps showing the distribution of Brn3a^+^RGCs in both mice strains, in two right and two left retinae. **(A′–H′)** Neighbor maps showing the distribution of ipRGCs in the same retina. At the bottom right of each map is shown the total number of cells counted. Color scale bar in **(A)** goes from 0 (purple) to the maximum (red) which is ≥4800 Brn3a^+^RGCs/mm^2^ for pigmented mice and ≥5625 Brn3a^+^RGCs/mm^2^ for albinos. The color scale in **(A’)** goes from 0 (purple) to 11 or more (red) neighbors in a radius of 0.165 mm. S: superior, T: temporal, I: inferior, N: nasal. Scale bar: 500 µm.

**Figure 5:**
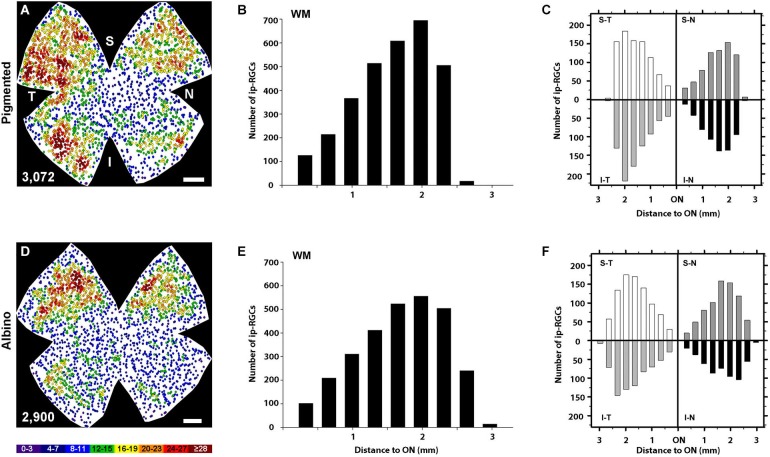
**Quantitative analysis of the retinal distribution of ipRGCs. (A,D)** Normalized neighbor maps where data from three pigmented **(A)** and three albino **(D)** retinas have been represented. Using these data, the number of ipRGCs at a given distance from the ON was plotted for the whole pigmented **(B)** and albino** (E)** retina, or for each of the four retinal quadrants in pigmented **(C)** and albino **(F)**. supero-temporal (ST), supero-nasal (SN), infero-temporal (IT) and infero-nasal (IN). At the bottom of each map is shown the number of ipRGCs represented. Color scale goes from 0–3 (purple) to ≥28 (red) neighbors. Scale bar: 500 µm.

Soma diameter measurement was performed using a IPP routine where soma perimeter was defined and diameter measurements were calculated as the average length of diameters at two degree intervals passing through cell body centroid.

Cross-sections were examined on the fluorescene microscope to count and identify the location of displaced ipRGCs, and to ascertain whether they were also OHSt^+^.

### Co-localization analyses

To determine the percentage of ipRGCs in the ganglion cell layer that were retrogradely labeled from the ON, from the SCi and/or that expressed Brn3a, photomicrographs of different representative areas were acquired for each signal. Images were overlapped with Adobe Photoshop (Adobe Photoshop CS 8.0.1; Adobe Systems, Inc., San Jose, CA, USA) and the percentage of ipRGCs that colocalized with OHSt and/or Brn3a was calculated, with 100% representing the total number of ipRGCs counted.

In the analysis of the retinal CMZ, the whole retinal perimeter was analyzed in the most intact (*n* = 3) retinae from mice in each experimental group and ipRGCs were identified by OHSt traced from the ON or SCi. To assess whether the d-ipRGCs were OHSt positive, the retina was carefully examined under the microscope to visualize each d-ipRGC, whose position we knew from the previous photomontage. Changing the filter to visualize the tracer or melanopsin fluorescence, each d-ipRGC was dotted on the retinal photomontage using a color-code representing all possible combinations (OHSt^+^d-ipRGCs or OHSt^−^d-ipRGCs).

For cross-sectional analysis, cryostat sections from two pigmented mice retinas that had been labeled with OHSt applied to the ON were examined to ascertain whether d-ipRGCs were also OHSt^+^ and to determine their location within the retinal layers.

### Statistics

Statistical analysis was done using SigmaStat^®^ 3.1 for Windows^®^ (SigmaStat^®^ for WindowsTM Version 3.11; Systat Software, Inc., Richmond, CA, USA). Kruskal–Wallis was used when comparing more than two groups and Mann–Whitney when comparing two groups only. For the comparison of ipRGC soma diameters between albino and pigmented mice, *t* tests were used unless otherwise stated. Differences were considered significant when *p* < 0.05.

## Results

### Identification of ipRGCs and Brn3a^+^ RGCs in retinal whole-mounts

In albino and pigmented rats (Nadal-Nicolás et al., 2012; Galindo-Romero et al., 2013) and in pigmented mice (Jain et al., 2012) Brn3a is expressed by the majority of RGCs and by a minute proportion of ipRGCs. Thus, double immunodetection of Brn3a and melanopsin is a good approach to study in the same retina, but independently, the general population of RGCs and ipRGCs. Therefore, in this work, melanopsin was always double immunodetected with Brn3a as shown in Figure 1. Although most of the ipRGCs were Brn3a negative (Figures 1A–D,Gb), co-localization analyses showed that in albino mice 0.78% of the ipRGCs were Brn3a^+^ (17 out of 2179 ipRGCs) while in the pigmented strain this proportion was 2.5 fold higher, (1.93%, 48 out of 2478 ipRGCs). Figure 1Ga shows a representative example of one of the few ipRGCs that co-expresses Brn3a.

As shown in Figure 1, the antibody employed in this study recognized ipRGCs of different size and staining intensity. The soma diameter of ipRGCs ranged from 8.5–21.4 µm (Table 2) and based on previous work (Berson et al., 2010; Estevez et al., 2012; Jain et al., 2012), our counts are most likely to include ipRGCs of the M1 and M2 subtype but may also include some of the less common M3 and larger M4 type cells.

**Table 2:** **Soma diameter of ipRGCs and d-ipRGCs in pigmented and albino mice**.

		Pigmented		Albino	
		ipRGCs *n* = 103	d-ipRGCs *n* = 69	ipRGCs *n* = 80	d-ipRGCs *n* = 30
Soma diameter (µm)	Mean ± SD	13.5 ± 1.8	11.8 ± 1.6	14.8 ± 2.2	12.9 ± 2.3
	Max	17.7	15.8	21.4	17.5
	Min	9.5	8.5	9.7	8.6

*Mean ± standard deviation (SD), maximum (Max) and minimum (Min) soma diameter (µm) of ipRGCs and d-ipRGC measured in three pigmented and three albino retinae. *n*: number of ipRGCs analyzed*.

### Retinofugal projections of ipRGCs

Neurons in the retina are classed as ganglion cells if they send their axon through the ON towards the brain. To verify that all melanopsin positive (M^+^) cells are indeed RGCs, colocalization of melanopsin and OHSt was analyzed in retinas traced from the ON (Table 3). As expected, almost 100% of the ipRGCs were traced. In this analysis, the whole retina was examined sparing the CMZ (see below).

**Table 3:** **ipRGCs project largely to the superior colliculi**.

	Tracing from	Number of ipRGCs analyzed	OHSt and melanopsin
Pigmented	SCi	1423	1401 (98.4%)
	ON	1055	1052 (99.7%)
Albino	SCi	1438	1398 (97.2%)
	ON	741	733 (98.9%)

*The percentage of colocalization is shown in brackets where 100% represents the total number of ipRGCs analyzed. SCi: superior colliculi. ON: optic nerve*.

The SCi are the main retinorecipient structures in rodents (Linden and Perry, 1983; Hofbauer and Dräger, 1985; Salinas-Navarro et al., 2009b,c; Nadal-Nicolás et al., 2014) and in rats, the majority of ipRGCs (90.62% as detected by melanopsin immunohistofluorescence) project to them (Galindo-Romero et al., 2013; Nadal-Nicolás et al., 2014). To understand whether this is also the case in mice, the proportion of OHSt positive ipRGCs was quantified in retinas traced from both SCi (Table 3). Our data shows that in both mice strains, the vast majority of the ipRGCs become retrogradely labeled when OHSt is applied to the SCi (See also Figures 1A,B,E,F,G).

### Retrograde tracing from the ON fails to label a subset of ipRGCs in CMZ

Recently it has been reported that in the CMZ of the C3H/He wildtype mouse retina, there exists a discrete plexus of ipRGCs with short melanopsin positive retino-ciliary projections (Semo et al., 2014). Thus, in order to determine if these melanopsin positive (M^+^) cells send axons into the ON towards the brain, we quantified/mapped the distribution of M^+^CMZ cells in both pigmented and albino mice following retrograde labeling with OHSt from either the ON or the SCi (Figures 2, 3).

In retinas traced from the ON, colocalization of melanopsin and OHSt was carefully examined in the CMZ from nasal and temporal hemiretina in both pigmented and albino mice (Figures 2A,B). As shown in the quantitative results (Figure 2C), the albino strain has, on average, almost double the number of M^+^ cells in the CMZ than the pigmented and this difference is statistically significant for the nasal retina (*p* = 0.003). Nevertheless, in both strains there are more M^+^ cells in the nasal CMZ (70% reside in nasal CMZ for pigmented mice and 66% reside nasally for albinos).

Surprisingly, in comparison to the whole retinal counts (Table 3), we found a much higher percentage (up to 25%) of M^+^ cells in the CMZ that were unlabeled by OHSt (M^+^OHSt^−^). As shown in Figure 2C, the proportion of M^+^OHSt^−^ to M^+^OHST^+^ cells was higher in the nasal vs. the temporal CMZ for both strains. The nasal predisposition for M^+^ cells is also illustrated in the representative plots of Figure 3.

Interestingly, the numbers of M^+^OHSt^−^ and M^+^OHST^+^ cells in the nasal and temporal CMZ were very similar between animals traced from the ON or SCi: for the nasal hemiretina of pigmented mice, ON tracing produced 26 ± 2 M^+^OHST^+^ and 8 ± 2 M^+^OHSt^−^ cells, while SCi tracing resulted in 24 ± 1 M^+^OHST^+^ and 9 ± 1 M^+^OHSt^−^ cells. For the nasal hemiretina of albinos, ON tracing produced 41 ± 13 M^+^OHST^+^ and 15 ± 4 M^+^OHSt^−^ cells, while SCi tracing produced 33 ± 4 M^+^OHST^+^ and 18 ± 1 M^+^OHSt^−^ cells. For the temporal hemiretina of pigmented mice, ON tracing produced 12 ± 4 M^+^OHST^+^ and 2 ± 0 M^+^OHSt^−^ cells, while SCi tracing gave 10 ± 3 M^+^OHST^+^ and 3 ± 1 M^+^OHSt^−^ cells. For the temporal hemiretina of albinos, ON tracing produced 20 ± 8 M^+^OHST^+^ and 6 ± 2 M^+^OHSt^−^ cells, while SCi tracing produced 19 ± 2 M^+^OHST^+^ and 6 ± 2 M^+^OHSt^−^ cells. We were unable to detect any significant differences between the numbers of M^+^ cells in the CMZ of mice traced from the ON and SCi. Figure 3 shows representative plots of M^+^CMZ cells in pigmented and albino retinae traced from either the ON or SCi.

This neuroanatomical tracing data strongly suggests that M^+^ cells in the CMZ of mice should be classified as either ipRGCs (M^+^OHST^+^) or M^+^OHST^−^ cells, the latter may be some type of interneuron or retino-ciliary projection neuron.

### Total number, size and spatial distribution of ipRGCs in the RGC layer

We quantified ipRGCs in the RGC layer of retinas with and without tracing. Automated quantification of OHSt^+^RGCs was obtained for retinas traced from the SCi. The data in Table 4 shows that the detection of ipRGCs is not affected by tracing.

**Table 4:** **Total number of ipRGCs and Brn3a^+^RGCs in naive retinae and in retinae traced from the superior colliculi**.

	Retinas	Brn3a^+^RGCs	ipRGCs	OHSt^+^RGCs
Pigmented	Traced from SCi (*n* = 7)	38,716 ± 2338	1069 ± 141	40,358 ± 2260
	No tracing (*n* = 12)	38,375 ± 1148	992 ± 78
	Mean	38,501 ± 1630	1021 ± 109
Albino	Traced from SCi (*n* = 10)	45,848 ± 3154	955 ± 163	46,577 ± 2479
	No tracing (*n* = 11)	45,907 ± 2547	968 ± 182
	Mean	45,884 ± 2707	962 ± 169

*Data are shown as mean ± standard deviation*.

As previously reported (Salinas-Navarro et al., 2009b), the albino strain has more RGCs than the pigmented one. With respect to Brn3a^+^RGCs, these are 95% and 98.5% of the projection to the SC in pigmented and albino mice, respectively. The ipRGCs accounted for 2.5% and 2.1% of the total OHSt^+^ traced population in pigmented and albino animals respectively. There was no statistically significant difference between total ipRGC numbers in pigmented or albino mice. We did however detect a statistically significant difference in the mean soma diameter of ipRGCs in pigmented (*n* = 103 cells, 13.5 ± 1.8 µm) vs. albino (*n* = 80 cells, 14.8 ± 2.2 µm) mice (Table 2, *t* test, *P* < 0.001). In terms of total ipRGC numbers counted, the 1021 ± 109 reported here for C57BL/6 mice is comparable to the 1194 ± 281 reported for the same strain in a recent study (Jain et al., 2012) but somewhat less than the 1600–1800 reported by Hughes et al. (2013).

The topography of ipRGCs and Brn3a^+^RGCs is shown in Figure 4. Here, Brn3a distribution is represented by isodensity maps (Figures 4A–H), while the distribution of ipRGCs is represented by neighbor maps (Figures 4A′–H′). In both strains, ipRGCs are more abundant in the periphery of the retina and they appear located in the areas of lower Brn3a^+^RGC density (See Figure 4 and quantitative analysis in Figures 5B,E).

However, there are subtle differences between strains, with pigmented mice showing a more highly populated temporal retina (Mann Whitney test, *p* = 0.001; Figures 5A,C), and albinos displaying more ipRGCs in the superior retina (Mann Whitney test, *p* = 0.001; Figures 4D,F). Furthermore, in the pigmented C57BL/6 mice, ipRGCs form a C like shape from the supero-temporal to the infero-temporal quadrant. In the albino mice, however, ipRGCs are denser in the superior retina forming an arc, above the highest Brn3a^+^RGC densities (Figures 4, 5). To date, the only other study to examine ipRGC distribution in the mouse retina showed a concentration of ipRGCs in the dorsal retina (Hughes et al., 2013). Our topography data in C57BL/6 mice differs somewhat from that reported by Hughes et al. (2013) using the same strain of mouse.

### Total number and spatial distribution of d-ipRGCs

**Figure 6:**
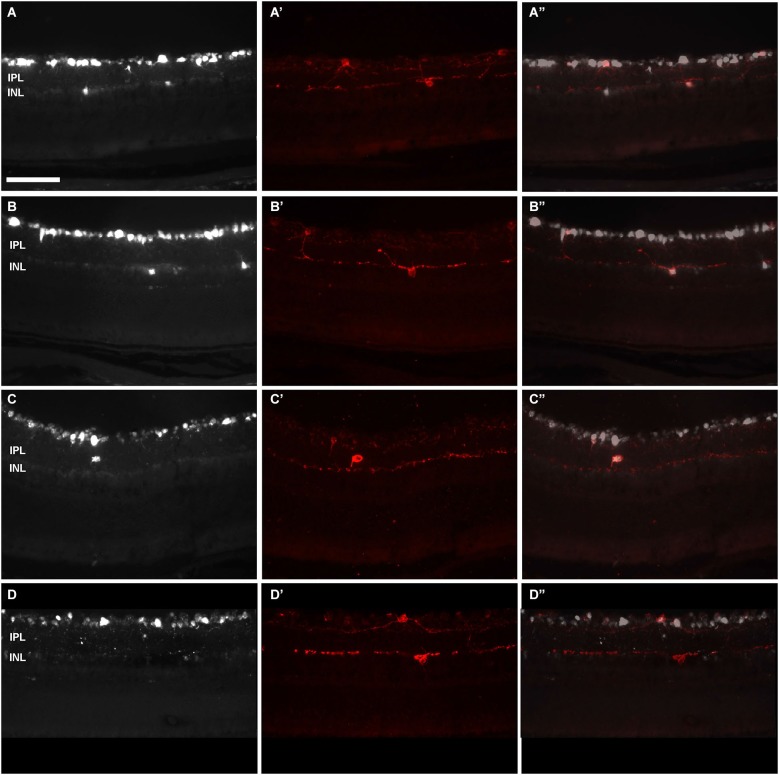
**Displaced-ipRGCs in retinal cross sections from pigmented mice**. Images acquired from retinal cross-sections showing OHSt **(A–D)** and melanopsin **(A’–D’)** signal. **(A”–D”)** are the corresponding merged images. Retinal ganglion cells were retrogradely labelled with OHSt from the SCi **(A–C)** or from the ON **(D)**. In **(A)** and **(B)** are observed OHSt^+^ displaced RGCs, out of which two are melanopsin positive **(A’,B’)**. As a rule, d-ipRGCs lay in the inner nuclear layer **(A–B”)**. After scrutinizing the sections from four retinas, one d-ipRGC was found in the inner plexiform layer **(C–C”)**. In **(D–D’)** is shown an example of a melanopsin positive cell that has not been traced with OHSt from the ON. This is a melanopsin interneuron. IPL: Inner plexiform layer. INL: inner nuclear layer. Scale bar: 100 µm.

**Figure 7:**
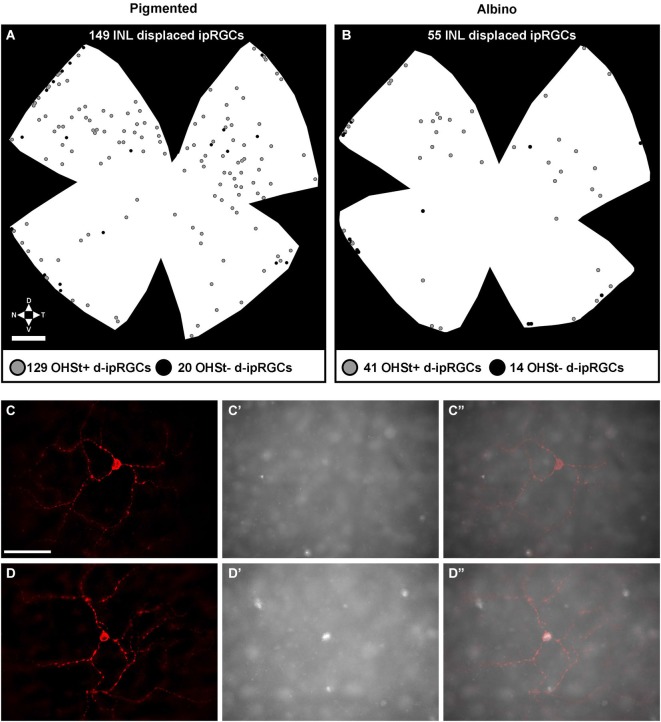
**Distribution of displaced ipRGCs (d-ipRGCs) in pigmented and albino retinae. (A,B)** Plots depicting the distribution of d-ipRGCs in a pigmented **(A)** and an albino **(B)** retina traced from the ON. Each dot represents a single d-ipRGC. Grey dots: d-ipRGCs traced from the ON (OHSt^+^). Black dots: d-ipRGCs that are not traced from the ON (OHSt^−^). **(C–D”)**: magnifications from a flat mounted retina traced from the ON where OHSt^−^ d-ipRGC **(C,C”)** and OHSt^+^ d-ipRGC **(D–D”)** are observed. **(C,D)** melanopsin signal, **(C’,D’)**: OHSt signal. **(C”,D”)**: merged images. Scale bar in **(A)** 500 µm in **(C)** 100 µm.

**Figure 8:**
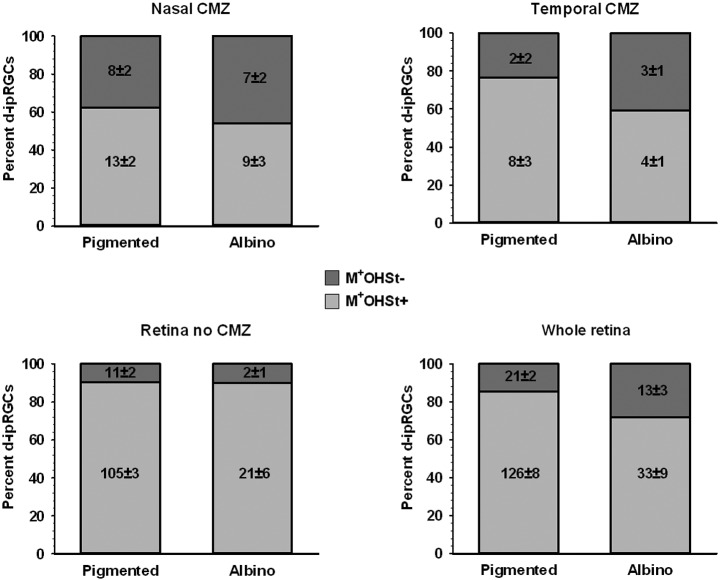
**Quantification of d-ipRGCs in the retinae of pigmented and albino mice traced from the ON**. Stacked bar graphs showing the total number (mean ± SD) and percentage of M^+^OHSt^+^ and M^+^OHSt^−^ d-ipRGCs in different regions of the pigmented and albino retina. Note that a relatively high percentage of d-ipRGCs in the CMZ are OHSt^−^ (top graphs) and that approximately half of the M^+^OHSt^−^ d-ipRGCs in pigmented mice are found in CMZ, while the majority of M^+^OHSt^−^ d-ipRGCs are located outside the CMZ in the albino retina (compare bottom two graphs).

In *n* = 4 eyes from pigmented animals retrogradely labeled with OHSt from the SCi, 15 µm thick frozen cryostat cross sections were examined for the location of d-ipRGCs. A total of 74 d-ipRGCs were identified in 91 sections analyzed, and everyone was found in the INL with the exception of one cell that was located in the IPL (Figure 6). In addition, cryostat sections from two pigmented mice retinas that had been labeled with OHSt applied to the ON were examined to ascertain whether d-ipRGCs were also OHSt^+^ and to determine their location within the retinal layers. In a total of 95 sections analyzed, 71 d-ipRGCs were identified in the INL with the exception of one cell that was located in the IPL, out of these 71 d-ipRGCs, 5 lacked OHSt labeling (Figure 6D). Displaced ipRGCs were quantified in *n* = 3 flat-mounted retinae from both pigmented and albino animals retrogradely labeled with OHSt from the ON. In both strains they comprised a small population of cells that were most numerous in dorsal retina (Figure 7). The d-ipRGCs could also be identified in the retinal CMZ, where they appeared most common nasally, with a slightly greater incidence of M^+^OHSt^−^ cells in the nasal hemiretina (Figure 8). In terms of mean soma diameter, the d-ipRGCs in both pigmented mice (*n* = 69 cells, 11.8 ± 1.7 µm) and albino mice (*n* = 30 cells, 12.9 ± 2.3 µm) were significantly smaller than their respective counterparts in the RGC layer (both *t* tests, *P* < 0.001).

The d-ipRGCs were far more common in pigmented C57BL/6 mice (mean ± SD of 147 ± 6.2) than albinos (46.3 ± 11) and accounted for ~14% and ~5% of their total ipRGC populations, respectively (for total ipRGC numbers see Table 4). They mainly appeared OHSt^+^, however, in comparison to conventional ipRGCs labeled from the ON (see Table 3), ~14% of the d-ipRGCs were unlabeled by OHSt in pigmented mice (mean of 21 ± 2 M^+^OHSt^−^), rising to ~28% (mean of 13 ± 3 M^+^OHSt^−^) in albinos. These cells appeared morphologically similar but we found it difficult to locate an obvious axon on M^+^OHST^−^ cells. As shown in Figure 8, for pigmented animals, the high percentage of M^+^OHSt^−^ d-ipRGCs may be accounted for in part by approximately half of these cells residing in the CMZ. However, this is not the case for M^+^OHSt^−^ d-ipRGCs in albino mice, most of which reside outside the CMZ (see bottom two graphs in Figure 8).

The disparity between retrograde co-localization data for conventional ipRGCs (located in the RGC layer) and d-ipRGCs (located in the INL) again raises the possibility that some d-ipRGCs fail to send an axon into the ON. As such, a small percentage of d-ipRGCs in the mouse retina may also in fact be interneurons and not RGCs.

## Discussion

Here we define the spatial distribution of melanopsin positive neurons in the retina of two commonly used mouse strains: the pigmented C57BL/6 mouse and the albino Swiss mouse. In addition to reporting important differences in ipRGC distribution between these two strains, we also describe that >97% of melanopsin expressing ipRGCs become retrogradely labeled when OHSt is applied to both SCi. Rather surprisingly, we also report the existence of melanopsin expressing neurons in the mouse retina which fail to label with a retrograde tracer applied to the ON. As such, we suggest that these cells are not ipRGCs and may instead constitute a new type of intrinsically photosensitive interneuron.

### ipRGCs population and its distribution in the retina

To date, only two other studies have reported total ipRGC counts in mouse retina, both also using the C57BL/6 strain (Jain et al., 2012; Hughes et al., 2013). The total ipRGC numbers we report here for pigmented mice are very similar to those reported by Jain et al. but less than the 1600–1800 reported by Hughes et al. Although all three studies use the same anti-melanopsin antibody, the difference in cell detection rate may have arisen due to the higher concentration used by Hughes et al. relative to the Jain study and our own work. Previous estimates of total ipRGC number in pigmented mouse retina range from ~1500 (Brown et al., 2010) to 2570 (Berson et al., 2010), although as stipulated by Berson et al., such estimates of total number are based on the assumption that ipRGCs are equally distributed across the retina.

As shown previously in rats (Nadal-Nicolás et al., 2012, 2014; Galindo-Romero et al., 2013), Brn3a was expressed by a very small percentage of ipRGCs. Together with other observations in mice (Jain et al., 2012), our analysis confirms that as in rats, Brn3a is an excellent marker for the concurrent visualization of ipRGCs and non-ipRGCs in mice. In terms of the topography of ipRGCs in the RGC layer, we show that the spatial distribution of these cells is more peripheral in both pigmented and albino mice, with the ipRGCs residing in regions of lower Brn3a^+^RGC density. Our analysis of ipRGC distribution revealed distinct differences between pigmented and albino mice, with the majority of ipRGCs found in the superior retina of albinos and the temporal hemiretina of pigmented mice.

To date, only one other study has examined the spatial distribution of ipRGCs in mice and this was also done using the C57BL/6 strain (Hughes et al., 2013). Although we used the same strain of mouse, our results differ from those of Hughes et al. who report that ipRGCs are most dense in the superior retina. However, one possible explanation for the discrepancy in our findings is based on the methods used to orientate mice retinae. Here we carefully orientate the mouse retina using several landmarks such as the rectus muscle insertion into the superior part of the eye and the nasal caruncle, and used the deepest cut to mark the superior pole of the eye (Salinas-Navarro et al., 2009b,c), while Hughes et al. do not use cuts (see Figure 1 and methods from Hughes et al., 2013), instead relying upon the distribution of UVS-opsin which is most intense in the ventral retina. Recent work from our laboratory using the C57BL/6 strain has shown that while UVS-opsin positive cones are indeed more common in the inferior retina, they show a marked predisposition for the nasal hemiretina (see Figures 1 and 4 of Ortín-Martínez et al., 2014). So, it may well be that the temporal retina is more heavily represented in the dorsal count data provided by Hughes et al. In support of a tempero-nasal gradient in ipRGC distribution, Hughes et al. do comment on a visually apparent “subtle variation” in ipRGC density between the lower retinal quadrants that did not reach significance using their analysis methods (see Figure S2 Hughes et al., 2013). This orientation issue should also be considered when interpreting the distribution of M4/M5 cells (Hughes et al., 2013), which may also reside more temporally.

Our finding that albino mice have significantly more RGCs than pigmented animals is in agreement with previous studies (Williams et al., 1996; Salinas-Navarro et al., 2009b). In addition, we also detected a significant difference in soma size and ipRGC distribution between the two strains. While no other studies to date have examined the topography of ipRGCs in albino mice, our data shows a similar superior distribution of ipRGCs to that found in albino rats. However, in albino rats, the predisposition for ipRGCs in the superior-temporal retina is more obvious (Hattar et al., 2002; Esquiva et al., 2013; Galindo-Romero et al., 2013; Nadal-Nicolás et al., 2014). These differences between pigmented and albino mice are consistent with a critical role for ocular pigmentation in the proper development of structure and function in rodents (Lund, 1965; Balkema and Dräger, 1990; Donatien and Jeffery, 2002). Together with observations of differences between the circadian regulation of melanopsin expression in pigmented and albino rats (Hannibal et al., 2005, 2013), we suggest that when studying the melanopsin system, caution should be exercised when interpreting data from albino animals.

### Displaced ipRGCs

In addition to ipRGCs in the RGC layer, mice, rats and primates also contain a population of d-ipRGCs in the INL. In mice, while these d-ipRGCs are known to show variable levels of immunoreactivity for melanopsin and Brn3b (Jain et al., 2012; Karnas et al., 2013), their spatial distribution is unknown. We show here that d-ipRGCs in both mouse strains studied were more common in the superior and far peripheral retina (i.e., CMZ). While previous estimates of the percentage of d-ipRGCs in C57BL/6 mice range between ~6% and ~9% (Berson et al., 2010; Jain et al., 2012; Karnas et al., 2013), our total cell count-derived figure of 14% includes the entire retina, including the CMZ. In albino mice, there were fewer d-ipRGCs (5%), a finding which agrees with the impact of pigmentation on both the general displaced RGC population in mice (Dräger and Olsen, 1980; Balkema and Dräger, 1990) and a recent comparison between d-ipRGC numbers in albino and pigmented rats (Nadal-Nicolás et al., 2014). Although the function of d-ipRGCs in mice is currently unclear, in primate/human retina they are very common indeed, ranging from 40–60% of the ipRGC population depending upon species studied (Dacey et al., 2005; Jusuf et al., 2007).

### ipRGCs projections

The results of our retrograde tracing experiment from SC reveal that >97% of ipRGCs become retrogradely labeled with OHSt applied to the surface of this structure. However, this conclusion comes with the caveat that our tracer application technique may also label ipRGC axons in the neighboring nuclei. Indeed, following close inspection of labeled brains (data not shown), we found that OHSt application to the SCi does indeed result in diffusion to neighboring nuclei that are also retinorecipient. These nuclei are the lateral geniculate (dorsal and ventral), intergeniculate and pretectal nuclei such as the NOT and OPN. Thus, the present technique does not allow us to discard the possibility that there may be separate sub-populations of ipRGCs projecting to the SC and neighboring pretectal and lateral geniculate nuclei. However, in our additional analyisis of brain sections, we could found no evidence that OHSt diffuses to the SCN, making it highly unlikely that this non-trans neuronal tracer would label ipRGCs projecting solely to the SCN.

There is a growing weight of evidence for a significant ipRGC input to the SC of mice. Firstly, this should be expected given that the vast majority of RGCs project to this structure in rodents (Linden and Perry, 1983; Hofbauer and Dräger, 1985; Salinas-Navarro et al., 2009b,c; Nadal-Nicolás et al., 2014). Also, anatomical studies in reporter mice which either preferentially label M1 cells (Hattar et al., 2006) or all ipRGC subtypes (Brown et al., 2010; Ecker et al., 2010) have both shown a substantial projection of ipRGC axons into superficial layers of SC (albeit it more extensively in the latter studies). While the less extensive input to SC in *tau-lacZ* mice has been taken as evidence that M1 cells do not significantly target this structure (Schmidt et al., 2011), it should be remembered that observations in *Opn4*^−/−^ mice may alternatively reflect a deficit in M1 axonal targeting to SC in the absence of melanopsin.

Interestingly, in the only other comprehensive retrograde tracing study to address central projections of M1 and M2 cells in *Opn4*^+/−^ mice (Baver et al., 2008), it was concluded that 100% of the ipRGCs projecting to the SC were of the M1 subtype (based on observations in *n* = 2 mice receiving small, localized injections). This same study found that 80% of M1 cells project to SCN, with approximately 45% of the input to OPN also emanating from the M1 subtype. Further evidence that M1 ipRGCs may project solely to the SCN comes from genetic ablation of the Brn3b positive population while maintaining SCN innervation and circadian rhythmicity (Chen et al., 2011). As our tracing data is likely to include the vast majority of M1 type ipRGCs, we propose that the SCN and OPN may simply be innervated by collaterals from retino-collicular ipRGC axons. This possibility has also been suggested before to explain the delayed postnatal innervation of the SCN by ipRGCs (McNeill et al., 2011). However, anterograde tracing in rats (Gooley et al., 2003) and retrograde tracing data in Hamsters (Morin et al., 2003) suggests that the SC may be a less prominent target in these species. Although, when interpreting the results of these studies, it should be appreciated that <50% of the melanopsin positive ipRGCs were labeled with rAAV-GFP by Gooley et al. (2003) and only one localized central region of SC was injected by Morin et al. (2003). Our results in mice may help to explain the strong light avoidance behavior in P6 mouse pups, which is the earliest recorded behavioral output of the melanopsin system, thought to be driven by the SC (Johnson et al., 2010).

Besides light avoidance behavior in mice, the significance of ipRGC input to the SC is at present unclear. It is known that the retino-collicular projection is topographically organized to provide a crude spatial map of the visual world (Siminoff et al., 1966). The SC is also well positioned to send visual information to both brainstem centers to alter eye movements and the basal ganglia to change visual attention/re-allocate motor resources (Comoli et al., 2003; May, 2006). As such, this structure would seem to be a prime candidate for mediating melanopsin-dependent orientation behavior in mice (Johnson et al., 2010; Semo et al., 2010) or perhaps even to initiate gaze/direct visual attention towards a brightly lit, emotionally salient target (Brown et al., 2012). So, the SC may either help to orientate animals towards bright and interesting targets or away from excessive levels of illumination (i.e., direct sun light). Along similar lines, in humans, ipRGC input to SC may contribute to the visual awareness seen in patients with rod/cone degeneration (Zaidi et al., 2007) and could also contribute to the subconscious aversive eye movements (squinting/averting gaze) that occur in response to direct sunlight (Sliney, 1997; Stringham et al., 2003). Interestingly, in support of this notion, there is now evidence of a significant ipRGC input to primate SC (Hannibal et al., 2014).

### Ciliary marginal zone (CMZ)

Recent work suggests that melanopsin positive cells in the retinal CMZ send projections into the ciliary body/iris to drive a component of the intrinsic pupillary light reflex (iPLR; Schmidt et al., 2014b; Semo et al., 2014). Given these findings, we wanted to confirm if all ipRGCs project an axon to the brain, and thus we applied OHSt onto the ocular stump of the intraorbitally transected ON (Salinas-Navarro et al., 2009b). As previously reported in C3H/He wildtype mice (Semo et al., 2014), we found a sub-population of M^+^cells in the CMZ of the nasal and to a lesser extent the temporal hemiretina of C57BL/6 and Swiss mice. In analysis that excluded the retinal CMZ, this procedure labeled 99.7% of all ipRGCs in the RGC layer of pigmented mice and 98.9% in the RGC layer of albinos, with ~90.5% and ~91.3% of d-ipRGCs labeled in pigmented and albino mice respectively. However, when the CMZ was analyzed in isolation the number of retrogradely labeled melanopsin positive cells fell to ~80% for pigmented mice and ~76% for albinos. We are not aware of any published data showing that subpopulations of neurons with an otherwise intact axon will fail to retrogradely transport the tracer we have used here. As such, this finding represents the first direct evidence that M^+^cells in the mammalian retina may not all be RGCs. It is thought that amphibians and fish posses a subset of melanopsin expressing horizontal cells (Provencio et al., 1998; Bellingham et al., 2002) and as such we suggest that the M^+^OHST^−^ cells reported here may be some type of intrinsically photosensitive retinal interneuron.

In the CMZ, such M^+^OHST^−^ cells may instead be sending their axon into the ciliary body/iris to elicit one component of the iPLR, as suggested previously (Semo et al., 2014). In other regions of the retina, there may also be a sub-population of M^+^OHST^−^ cells in the INL which could be engaged in the intra-retinal signaling phenomenon reported by others (Zhang et al., 2008). In support of our tracing data, we could not locate obvious axons in the OHSt^−^d-ipRGC cells. Similar issues have been raised by others trying to identify melanopsin positive processes arising from the d-ipRGCs of mice (Karnas et al., 2013) and axons were apparently not always visible in the X-gal stained d-ipRGCs of *tau-lacZ* reporter mice (Hattar et al., 2006). Our finding of M^+^OHST^−^ cells in the mouse retina has important implications for the much larger population of ipRGCs residing in the INL of primate retina which are most common centrally (Dacey et al., 2005; Jusuf et al., 2007). Interestingly, Jusuf et al. (2007) also report an increase in ipRGC density in the far nasal periphery of the macaque and marmoset retina, a finding which may correspond to the CMZ population reported in mice (Semo et al., 2014).

The melanopsin positive cells in mouse CMZ are largely Brn3b negative (Semo et al., 2014) and as such would be expected to project to SCN (Chen et al., 2011). However, in terms of comparison between retinas retrogradely traced from SCi and ON, we find it highly unlikely that any of them do project to SCN. This is because we could find no significant difference between numbers of traced and untraced melanopsin cells in the SCi-traced and ON-traced groups. So, it appears that M^+^cells in the CMZ of mice either project to the dorsal midbrain (SC and/or OPN) or fail to send an axon along the ON at all. Interestigly, anatomical tracing work has also shown that displaced RGCs are unlikely to project to the SCN in mice (Balkema and Dräger, 1990), so it may be that the M1 type of d-ipRGC also fail to project to SCN, perhaps instead being involved in other, non-circadian melanopsin-based functions such as the PLR.

In summary, the present study extends our understanding of the distribution of ipRGCs in mice and provides important evidence that not all ipRGCs project axons into the ON. We report that ipRGCs are more common in peripheral and temporal regions of the pigmented mouse retina and also show that the vast majority of ipRGCs become retrogradely labeled with OHSt applied to both SCi. This data may be important to consider in terms of an emerging role for melanopsin in brightness discrimination and image forming vision (Brown et al., 2010; Ecker et al., 2010; Allen et al., 2014; Schmidt et al., 2014a).

## Authors and contributors

All authors have reviewed and approved the final version of this work.

Conceptualized and designed the experiments: Francisco J. Valiente-Soriano, Diego García-Ayuso, Arturo Ortín-Martínez, Manuel Jiménez-López, Caridad Galindo-Romero, Maria Paz Villegas-Pérez, Marta Agudo-Barriuso, Anthony A. Vugler, Manuel Vidal-Sanz.

Performed the experiments: Francisco J. Valiente-Soriano, Diego García-Ayuso, Arturo Ortín-Martínez, Caridad Galindo-Romero.

Data acquisition: Francisco J. Valiente-Soriano, Diego García-Ayuso, Arturo Ortín-Martínez, Manuel Jiménez-López, Caridad Galindo-Romero.

Data analysis: Francisco J. Valiente-Soriano, Diego García-Ayuso, Arturo Ortín-Martínez, Manuel Jiménez-López, Caridad Galindo-Romero, Maria Paz Villegas-Pérez, Marta Agudo-Barriuso, Anthony A. Vugler, Manuel Vidal-Sanz.

Design of automated routines: Manuel Jiménez-López.

Data interpretation, manuscript drafting: Francisco J. Valiente-Soriano, Diego García-Ayuso, Arturo Ortín-Martínez, Manuel Jiménez-López, Caridad Galindo-Romero, Maria Paz Villegas-Pérez, Marta Agudo-Barriuso, Anthony A. Vugler, Manuel Vidal-Sanz.

Contributed reagents/materials/analysis tools: Maria Paz Villegas-Pérez, Marta Agudo-Barriuso, Anthony A. Vugler, Manuel Vidal-Sanz.

## Conflict of interest statement

The authors declare that the research was conducted in the absence of any commercial or financial relationships that could be construed as a potential conflict of interest.
